# Small Cell Lung Cancer Associated With Cystic Airspace: A Case Report and Literature Review

**DOI:** 10.1002/rcr2.70709

**Published:** 2026-08-02

**Authors:** Hiroyuki Shimada, Yukihisa Inoue, Osamu Matsubara, Mio Yamamoto, Shohei Yamashita, Tetsu Hara, Kazuki Yamanaka, Yasuto Jinn

**Affiliations:** ^1^ Department of Respiratory Medicine Hiratsuka Kyosai Hospital Hiratsuka Japan; ^2^ Department of Diagnostic Pathology Hiratsuka Kyosai Hospital Hiratsuka Japan; ^3^ Department of Surgery Hiratsuka Kyosai Hospital Hiratsuka Japan

**Keywords:** cystic airspace, lung cancer, small cell lung cancer

## Abstract

Cystic airspaces are recognised as a risk factor for lung cancer. Nearly all cancers arising in association with cystic airspaces are non‐small cell lung cancers, whereas cases of small cell lung cancer (SCLC) in this setting are rarely reported. A 72‐year‐old woman with a history of smoking was referred to our hospital for evaluation of an abnormal chest shadow. Computed tomography (CT) revealed a cystic airspace in the left upper lobe, along with a nodular lesion adherent to the cyst wall with polypoid extension into the lumen. Marked uptake of 18F‐fluorodeoxyglucose was observed in the nodular lesion on positron emission tomography–CT. Consequently, primary lung cancer was suspected, and surgical resection was performed. Histopathological examination of the resected specimen led to a diagnosis of SCLC. Although rare, SCLC could arise from cystic airspaces. Nodules abutting cystic airspaces, as well as cyst wall thickening, warrant close short‐term follow‐up.

## Graphical abstract

We report a rare case of small cell lung cancer (SCLC) associated with a cystic airspace. Chest CT revealed a solid tumour component arising from the wall of a pre‐existing lung cyst. This case highlights the clinical importance of recognising cystic airspaces as potential sites for aggressive malignancies like SCLC.

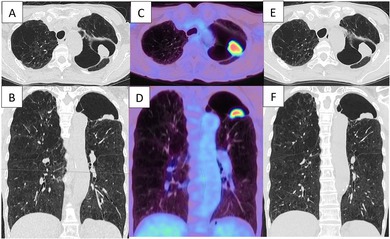

## 1 Introduction

Small cell lung carcinoma (SCLC) is a highly malignant neuroendocrine carcinoma that is strongly associated with smoking history. It accounts for approximately 15% of all lung cancers. There is no universally accepted staging system for SCLC. It is commonly classified into limited‐stage disease (LS‐SCLC), in which the tumour is confined to one hemithorax and can be encompassed within a tolerable radiation field, and extensive‐stage disease (ES‐SCLC), in which the tumour spreads beyond these limits. Compared with other histological types of lung cancer, there are fewer therapeutic options, and the prognosis is poor, with 5‐year survival rates of 12.0% for ES‐SCLC and 16.1%–27.7% for LS‐SCLC, respectively [1].

Cystic airspaces are recognised as a risk factor for lung cancer. However, lung cancers associated with cystic airspaces (LCCA) are relatively uncommon. Previous studies have reported that 3.7% of lung cancers detected on computed tomography (CT) are associated with cystic airspaces [2]. LCCA are often difficult to distinguish from benign lesions such as infectious diseases and may therefore be misdiagnosed, resulting in failure to detect them at an early stage [3]. Regarding the histological subtypes, it has been reported that in a series of 341 LCCA cases, adenocarcinoma was the predominant subtype (88.1%), while no cases of SCLC were identified [4]. SCLC associated with cystic airspaces is therefore exceedingly rare, with only three cases reported to date [3, 5, 6]. We report a case of the rapidly growing nodule arising from a cystic airspace that was surgically resected and diagnosed as LS‐SCLC.

## 2 Case Report

**FIGURE 1:**
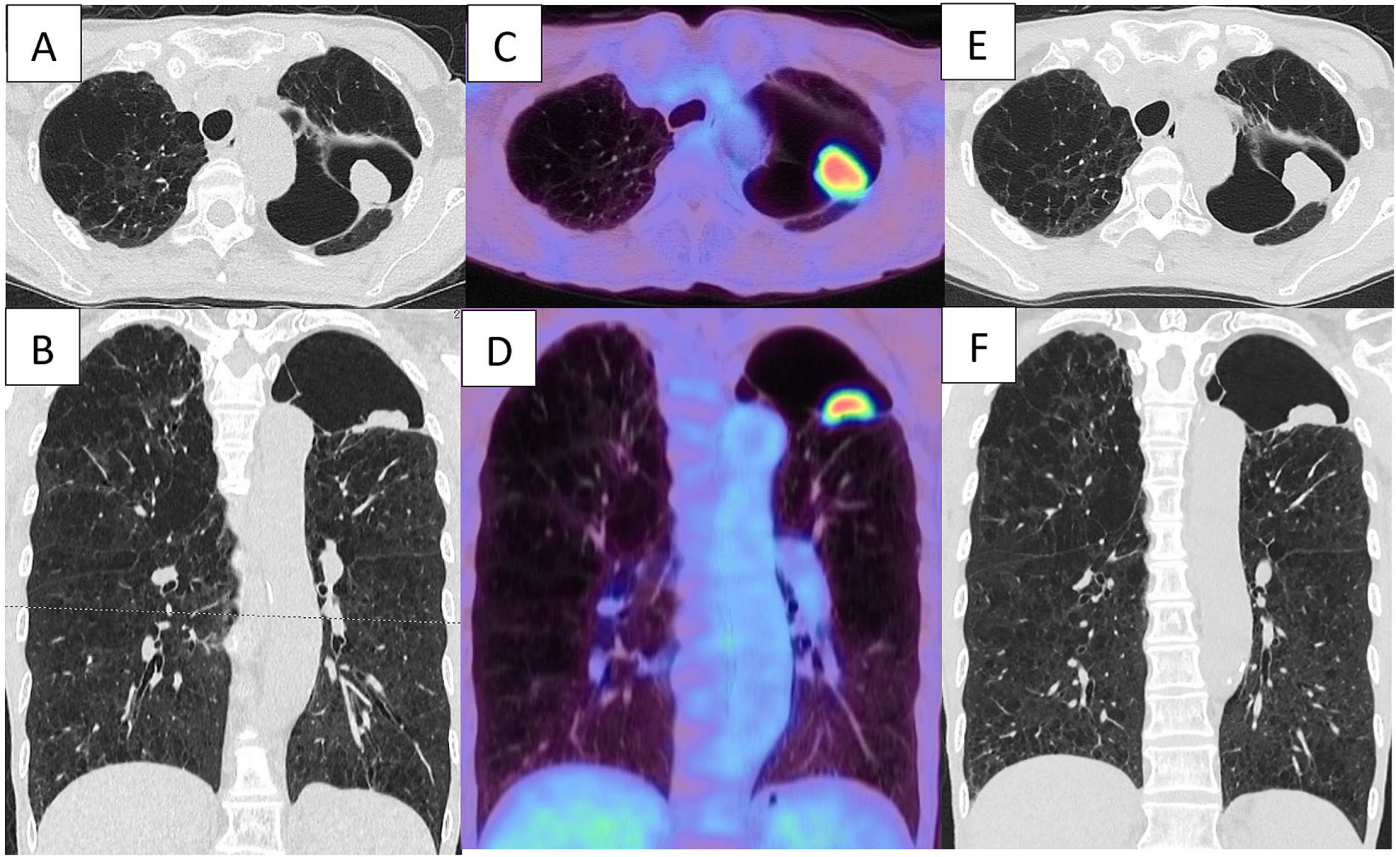
Computed tomography and 18F‐fluorodeoxyglucose positron emission tomography images. (A, B) At the initial visit, the nodule protruding into a cyst was observed in the left upper lobe. (C, D) The nodule showed increased uptake with a maximum standardised uptake value of 5.5. (E, F) The nodule showed growth at 1‐month follow‐up.

A 72‐year‐old woman with a 25 pack‐year smoking history, who was asymptomatic, presented to our hospital in July 2025 for further evaluation of an abnormal chest shadow. Several years earlier, a cystic lesion had been noted in the left upper lung field on chest radiography during health screening, but no further evaluation was performed. Chest x‐rays showed a new nodular lesion adherent to the cyst wall, prompting referral to our hospital for further evaluation with chest CT. CT revealed a 74 × 84 × 53 mm cystic airspace in the left upper lobe, along with a 23 × 24 × 10 mm nodular lesion adherent to the cyst wall with polypoid extension into the lumen (Figure 1A,B). Physical examination revealed no abnormalities. Laboratory findings showed an elevated serum pro–gastrin‐releasing peptide (ProGRP) level (163 pg/mL), there was no elevation in white blood cell count or serum C‐reactive protein levels. Additionally, both the serum Aspergillus antigen and antibody tests were negative. 18F‐fluorodeoxyglucose positron emission tomography demonstrated increased uptake in the nodule, with a maximum standardised uptake value of 5.5 (Figure 1C,D).

**FIGURE 2:**
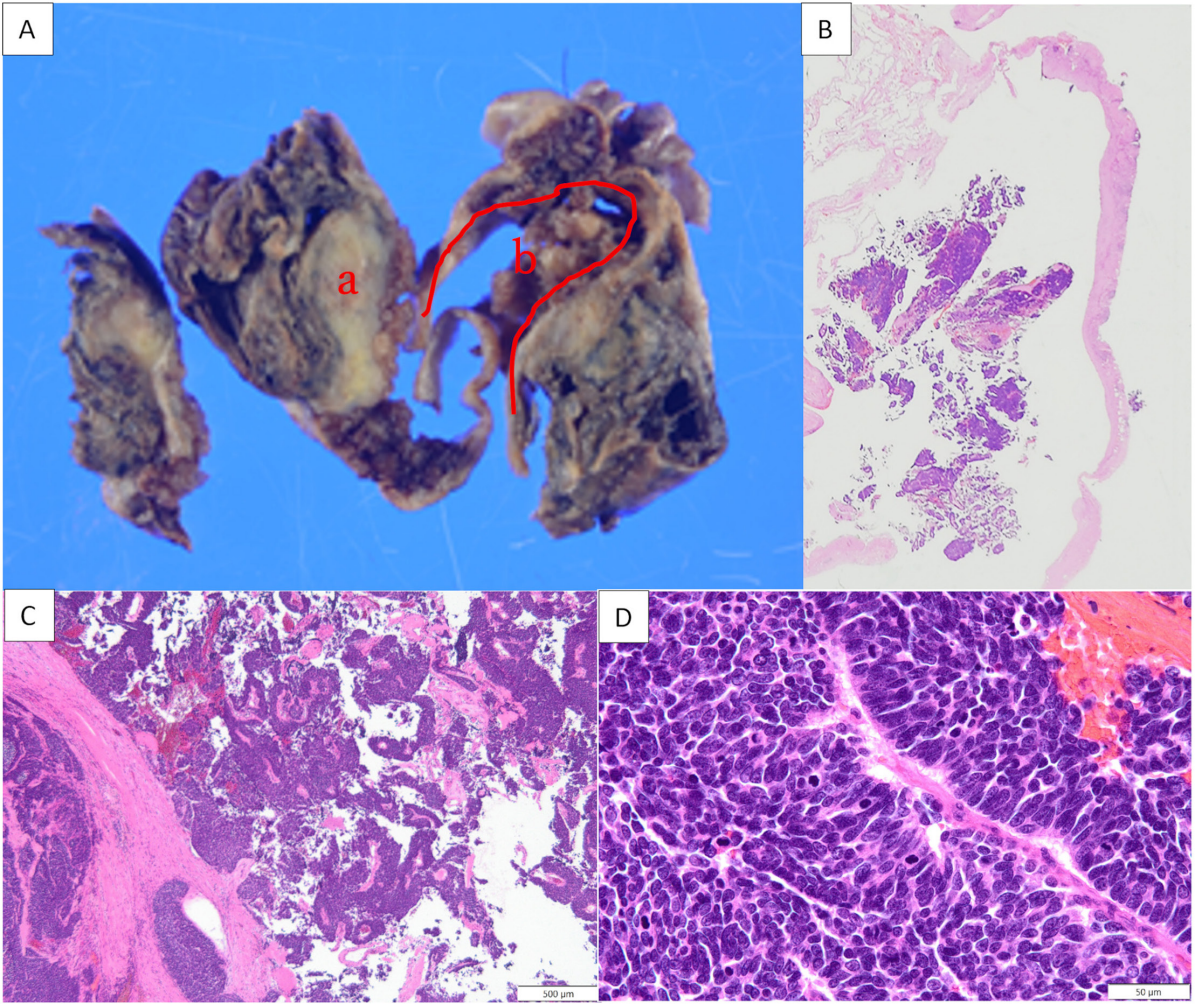
Pathological findings. (A) Macroscopic findings. A grey‐white mass (a) was identified adherent to the cyst wall (the red line around the label ‘b’). (B) Photograph of the glass slide, showing a tumour protruding into the cyst cavity. (C, D) Microscopic findings. (C) Low magnification view showing dense proliferation of tumour cells. (D) High magnification view revealing small atypical cells with a high nuclear‐to‐cytoplasmic ratio and numerous mitotic figures, consistent with small cell carcinoma.

A follow‐up CT after 1 month demonstrated growth of the lesion to 31 × 28 × 12 mm (Figure 1E,F). Therefore, the likelihood of malignancy was considered higher. If the lesion had represented lung cancer, it would have been clinically classified as cT2aN0M0 (stage IB). As the lesion was difficult to access bronchoscopically, bronchoscopic evaluation was not feasible. Surgical resection was proposed for both diagnostic and therapeutic purposes. The patient opted for surgery after fully understanding that a benign lesion could not be ruled out. Preoperative examination revealed a decreased FEV_1_/FVC ratio, which remained low at 65.9% even after bronchodilator treatment. A limited resection was planned, and a thoracoscopic partial resection of the left upper lobe was performed in October 2025. After plication of the cystic airspace, both the cystic airspace and the tumour were resected with an adequate surgical margin. The surgery was completed as planned without complications. In the resected specimen, a grey‐white mass measuring 35 mm in maximum diameter was identified adherent to the cystic airspace wall (Figure 2A,B). The cystic airspace was a typical cyst characterised by fibrous wall thickening. Fibrous scarring and calcified foci were observed around the base of the tumour in the cyst wall. The tumour was compatible with small cell carcinoma, consisting of densely packed atypical polygonal to oval cells with a high nuclear‐to‐cytoplasmic ratio (Figure 2C,D). The tumour cells were positive for neural cell adhesion molecule (CD56), synaptophysin, insulinoma‐associated protein 1 (INSM1), and thyroid transcription factor‐1 (TTF‐1) on immunohistochemistry. Histological examination revealed focal discontinuous tumour lesions within the lung, which were diagnosed as intrapulmonary metastases. A key characteristic distinguishing LCCA from conventional cavitary lung cancer is that the cystic airspace is larger than the associated solid or non‐solid tumour components. As the present case fulfilled this criterion, the patient was diagnosed with SCLC associated with a cystic airspace, pT3N0M0, stage IIB (LS‐SCLC), based on the pathological findings and preoperative imaging. Postoperative adjuvant chemotherapy with carboplatin and etoposide were administered, and no recurrence has been observed until present follow‐up extending 10 months after surgery.

## 3 Discussion

Cystic airspaces in the lung are associated with an increased risk of lung cancer, and several mechanisms have been proposed to explain this association. The following mechanisms have been proposed, associations with fibrosis and scarring (so‐called scar cancer), squamous metaplasia, particularly in squamous cell carcinoma, and accumulation of carcinogenic substances within cysts, creating a precancerous environment [7]. In the present case, fibrotic scarring and focal calcifications were observed in the cyst wall adjacent to the tumour. Tumour may have arisen via a scar carcinoma mechanism associated with pre‐existing inflammatory scarring. Additionally, SCLC is thought to originate primarily from neuroendocrine cells following inactivation of the tumour suppressor genes RB1 and TP53 [8]. Clara cells and alveolar type II cells may also serve as less frequent cells of origin under the same genetic alterations [9]. These cells proliferate and differentiate during the repair of injured bronchial and alveolar epithelium. In the present case, they may have been increased within the inflamed cyst wall and served as the site of origin for SCLC, while smoking‐related accumulation of carcinogens within the cyst may have induced DNA damage in RB1 and TP53, thereby contributing to tumour development. Although linking the development of SCLC to both cyst wall inflammation and the accumulation of carcinogens represents one possible mechanism in this case, this hypothesis does not explain why SCLC is extremely rare among LCCA. A review of the literature identified only three previously reported cases of SCLC associated with cystic airspaces (Table 1). All cases had a smoking history and were complicated by emphysematous lung disease. Many cases presented with SCLC in the left upper lobe. While approximately 70% of SCLC cases are typically diagnosed as ES‐SCLC [1], three out of the four cases in this study were diagnosed with LS‐SCLC, suggesting a trend towards earlier detection. To clarify the characteristics of SCLC among LCCA, further accumulation of cases is warranted.

**TABLE 1:** Reported cases of small cell lung cancer associated with cystic airspaces.

References	Age	Sex	Smoking history	Comorbidities	Reason for presentation	Site of origin	Primary tumour size	Cancer staging	LCCA classification^a^	Treatment	Clinical course
Buchanan et al. [5]	62	M	Yes	Pneumothorax, pulmonary emphysema	Chest pain (due to pneumothorax)	LUL	20 mm	LS‐SCLC	Type III	Surgery, adjuvant chemotherapy and prophylactic cranial irradiation	Alive ≥ 2 years without recurrence
Seike et al. [6]	71	M	Yes	Pulmonary emphysema	Headache (due to brain metastases)	LUL	NR	ES‐SCLC	NR	Chemoimmunotherapy	Alive ≥ 3 years without recurrence
Touge et al. [3]	68	M	Yes	COPD	Abnormal chest findings	RLL	8 mm	LS‐SCLC	Type I	Chemotherapy	Alive ≥ 2 years with recurrence
Our case	72	F	Yes	COPD	Abnormal chest findings	LUL	35 mm	LS‐SCLC	Type II	Surgery and adjuvant chemotherapy	Alive ≥ 10 months without recurrence

Abbreviations: COPD, chronic obstructive pulmonary disease; ES‐SCLC, extensive‐stage small cell lung cancer; LS‐SCLC, limited‐stage small cell lung cancer; LUL, left upper lobe; NR, not reported; RLL, right lower lobe.

^a^ According to Mascalchi et al. classification of lung cancers associated with cystic airspaces (LCCA).

Mascalchi et al. classified LCCA into four types [10]. Type I features a solid nodule protruding externally from the cyst wall, while Type II shows one protruding internally. Type III involves circumferential thickening of the cyst wall, and Type IV presents tissue intermixed within clusters of cysts, with Types III and IV being more common [10]. This case represents the relatively rare Type II subtype, whereas previously reported cases of SCLC associated with cystic airspaces have been classified as Types I and III [3, 5]. However, the clinical significance of this four‐type classification remains unclear, including its features, prognosis, and association with histological subtypes. LCCA is often difficult to distinguish from benign lesions such as infections based on CT findings and is frequently misdiagnosed [3]. While PET scans can aid in diagnosis, some cases demonstrate poor uptake in the early stage [10]. When nodular lesions adjacent to cystic airspace walls or cystic wall thickening are observed, careful follow‐up is required even if the lesion is small.

While infrequent, the possibility of SCLC developing from cystic airspaces should not be overlooked. Consequently, close short‐term follow‐up is essential for any nodular lesions or wall thickening associated with cystic airspaces.

## Author Contributions

Hiroyuki Shimada and Yukihisa Inoue managed the patient, collected the clinical data, and drafted the manuscript. Osamu Matsubara performed the histological examinations and prepared the pathological figures. Mio Yamamoto, Shohei Yamashita and Tetsu Hara reviewed the literature, revised the manuscript for intellectual content. Kazuki Yamanaka managed the patient, collected the clinical data. Yasuto Jinn reviewed the literature, revised the manuscript for intellectual content, and gave final approval for publication. All authors have read and agreed to the published version of the manuscript.

## Consent

The authors declare that written informed consent was obtained for the publication of this manuscript and accompanying images using the consent form provided by the Journal.

## Conflicts of Interest

The authors declare no conflicts of interest.

## Data Availability

Data sharing not applicable to this article as no datasets were generated or analysed during the current study.
